# Supplementary material for: The extra-terminal domain drives the role of BET proteins in transcription
Source: Nucleic Acids Res. 2026 Jul 16;54(13):gkag717. doi: 10.1093/nar/gkag717 (PMC13373324; doi:10.1093/nar/gkag717)
Supplement: gkag717_Supplemental_Files [file gkag717_supplemental_files.zip › Pasionek_etal_2026_Supplementary.pdf]

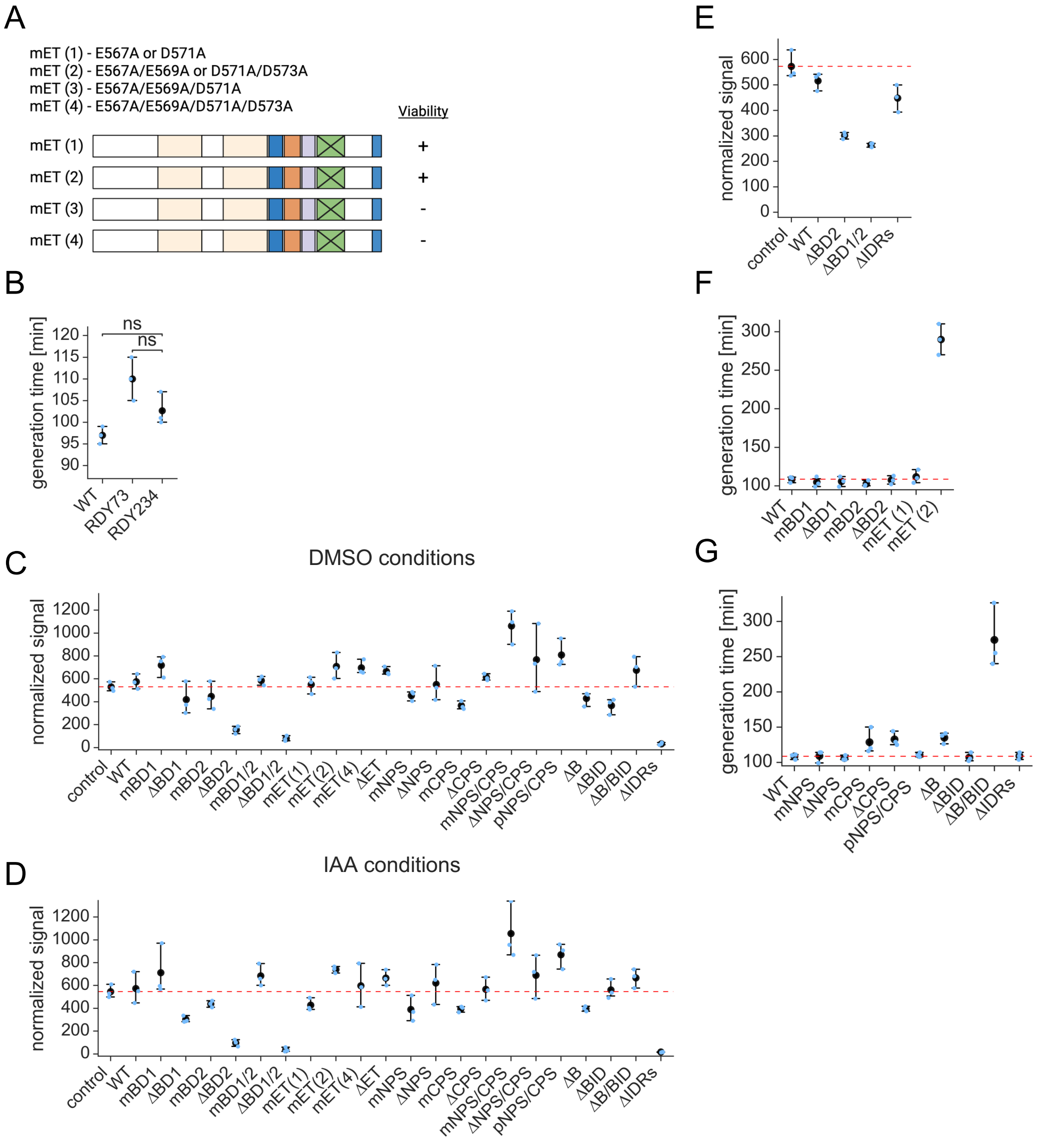

Fig. S1. At least two conserved acidic residues in the ET domain are required for viability. (**A**) Six Bdf1 variants carrying targeted mutations in the ET domain. Ability to support growth in the absence of endogenous *BDF1/2* is indicated. (**B**) Growth assay comparing the fitness of a laboratory wild-type strain (BY4705, WT), the RDY73 strain, and an RDY73 derivative expressing additional copy of unmodified *BDF1* from the *TRP1* locus (RDY234). Black markers indicate mean generation times. Error bars represent the 95% confidence interval (n = 3). Results of a Welch's t-test are shown (ns – not significant). (**C**) Western blot analysis of the expression of the indicated *BDF1* variants from the *TRP1* locus of strain RDY73 without depletion of endogenous Bdf1/2 (DMSO conditions). The reference expression level (red dashed line) corresponds to the parental strain BY4705. An anti-Bdf1 antibody was used (a gift from Stephen Buratowski). Fluorescent signal for total protein content (LI-COR total protein stain workflow) was used to normalize target proteins. Black markers indicate mean normalized signal. Error bars represent the 95% confidence interval (n = 3). (**D**) Same as (**C)** but with depletion of endogenous Bdf1/2 (IAA conditions) (n = 3). (**E**) Western blot analysis of the expression of the indicated *BDF1* variants carrying 3xFLAG epitope. *BDF1* variants were expressed from the *TRP1* locus of strain RDY73 without depletion of endogenous Bdf1/2. The

reference expression level (red dashed line) corresponds to the control strain expressing Bdf1-3xFLAG from the endogenous locus. An anti-FLAG antibody (Sigma, F3165) was used. Fluorescent signal for total protein content (LI-COR total protein stain workflow) was used to normalize target proteins. Black markers indicate mean normalized signal. Error bars represent the 95% confidence interval (*n* = 3). (**F-G**) Growth assays comparing the fitness of a control strain expressing unmodified *BDF1* (WT) to strains expressing *BDF1* variants after 15 h exposure to IAA (long-term loss of endogenous Bdf1/2). Black markers indicate mean generation time. Error bars represent 95% confidence intervals (*n* = 3).

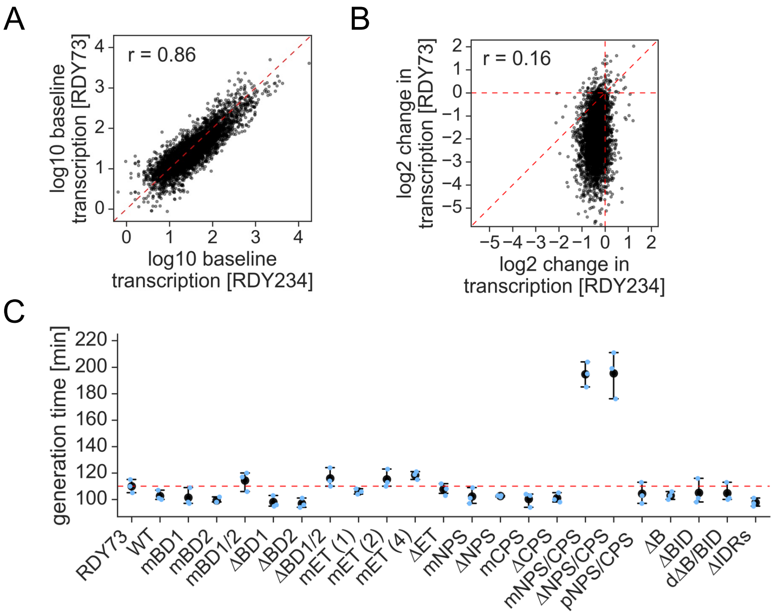

Fig. S2. Additional copy of *BDF1* is well tolerated by yeast cells. (**A**) Comparison of baseline transcription (log10) in strains RDY73 (Bdf1/2-AID) and its derivative expressing an additional copy of unmodified *BDF1* from the *TRP1* locus (RDY234). Pearson correlation coefficient (r) is shown (n = 4,836). (**B**) Log2 changes in transcription measured by SLAM-seq after depleting endogenous Bdf1/2 for 25 min in strains RDY73 and RDY234. Pearson correlation coefficient (r) is shown (n = 4,836). (**C**) Growth assay comparing the fitness of strains expressing unmodified Bdf1 (WT) or the indicated Bdf1 variants to the parental strain RDY73 in the presence of endogenous Bdf1/2 (no IAA treatment). Black markers indicate mean generation time. Error bars correspond to 95% confidence interval (n = 3).

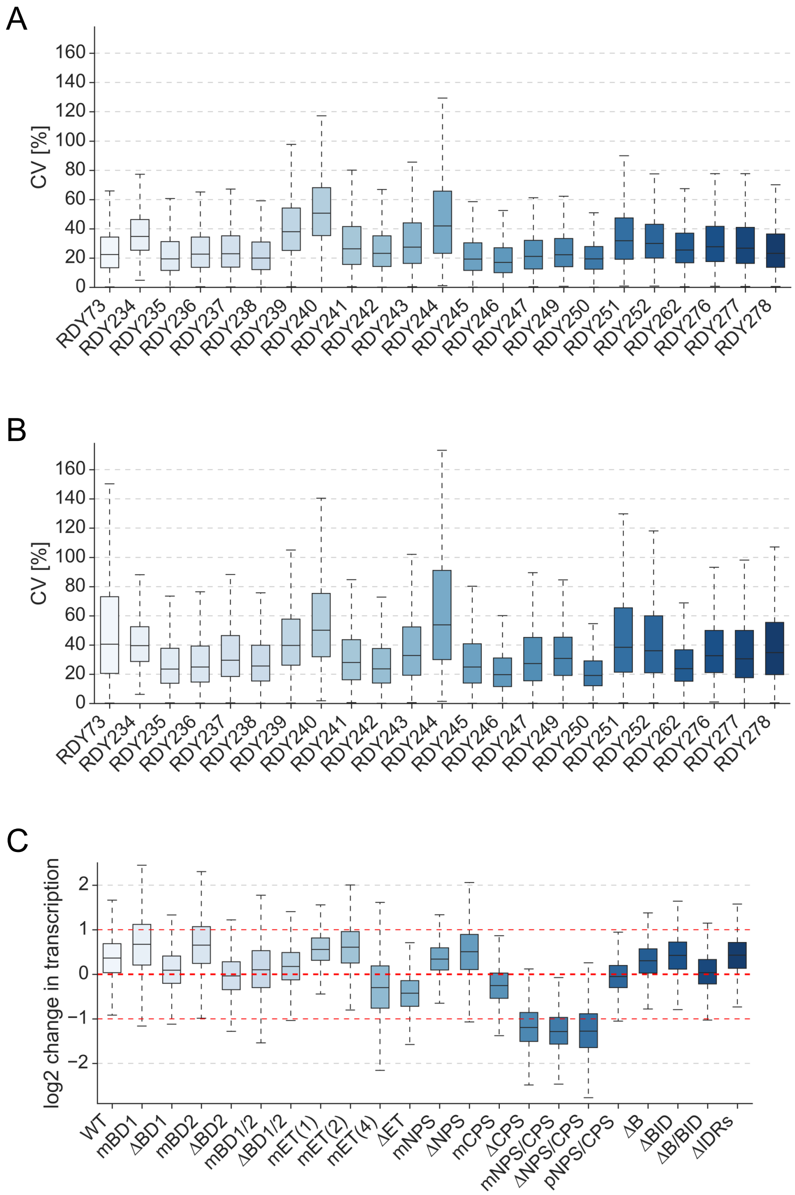

Fig. S3. SLAM-seq accurately captures rapid changes in transcription resulting from targeted mutations in BET protein domains. (**A**) Coefficient of variation (CV) for the indicated SLAM-seq experiments under DMSO conditions. CV values were calculated for each gene based on normalized read counts from replicate experiments (**Table S3**) (*n* = 4,836). (**B**) Coefficient of variation (CV) for the indicated SLAM-seq experiments under IAA conditions. CV values were calculated for each gene based on normalized read counts from replicate experiments (**Table S3**) (*n* = 4,836). **(C)** Log2 changes in transcription due to *BDF1* mutations without depletion of endogenous Bdf1/2 (*n* = 4,836).

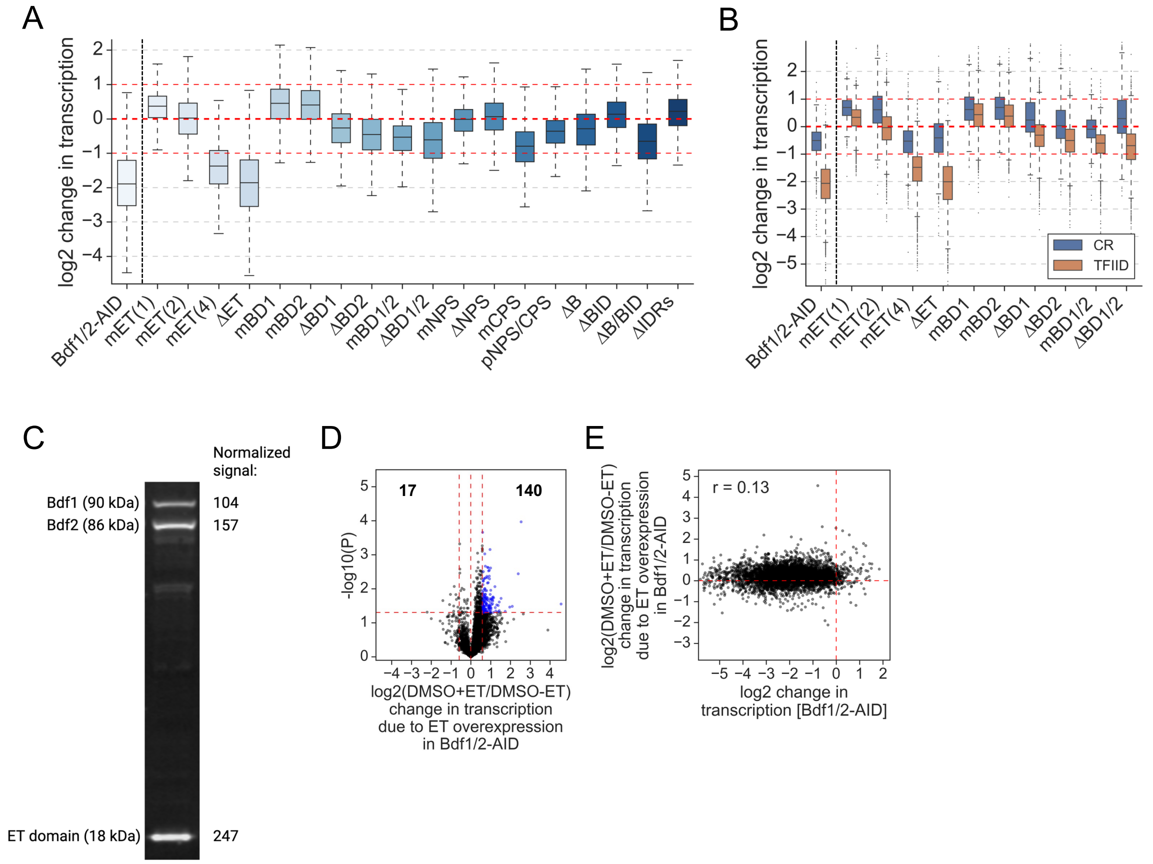

Fig. S4. The ET domain is the only BET protein domain broadly required for transcription. (**A**) Log2 changes in transcription due to *BDF1* mutations measured by SLAM-seq after depleting endogenous Bdf1/2 for 25 min (*n* = 4,836). Results of depletion of Bdf1/2 in the absence of an additional copy of *BDF1* are shown in the first column from the left. (**B**) Log2 changes in transcription due to *BDF1* mutations measured by SLAM-seq after depleting endogenous Bdf1/2 for 25 min. Genes are divided into previously defined major yeast gene classes (CR – coactivator-redundant, TFIID – TFIID-dependent) (68) (*n* = 4,560). (**C**) Western blot analysis of the ET domain overexpression in the RDY73 derivative strain RDY388. An anti-V5 antibody was used to detect target proteins. Fluorescent signal for total protein content (LI-COR total protein stain workflow) was used to normalize target proteins. A ~13-fold ET domain overexpression compared to Bdf1 was observed after correcting for the size difference. A representative image of three biological replicates is shown. (**D**) Volcano plot comparing transcriptional changes due to ET domain overexpression (log2 scale) in the presence of endogenous Bdf1/2 with the associated *p*-value. Thresholds used: fold change – 1.5-fold, *p*-value – 0.05 (Welch's t-test). (**E**) Comparison of transcriptional changes due to ET domain overexpression in the presence of endogenous Bdf1/2 (y-axis) with gene dependence on Bdf1/2 (x-axis). Pearson correlation coefficient (r) is shown.

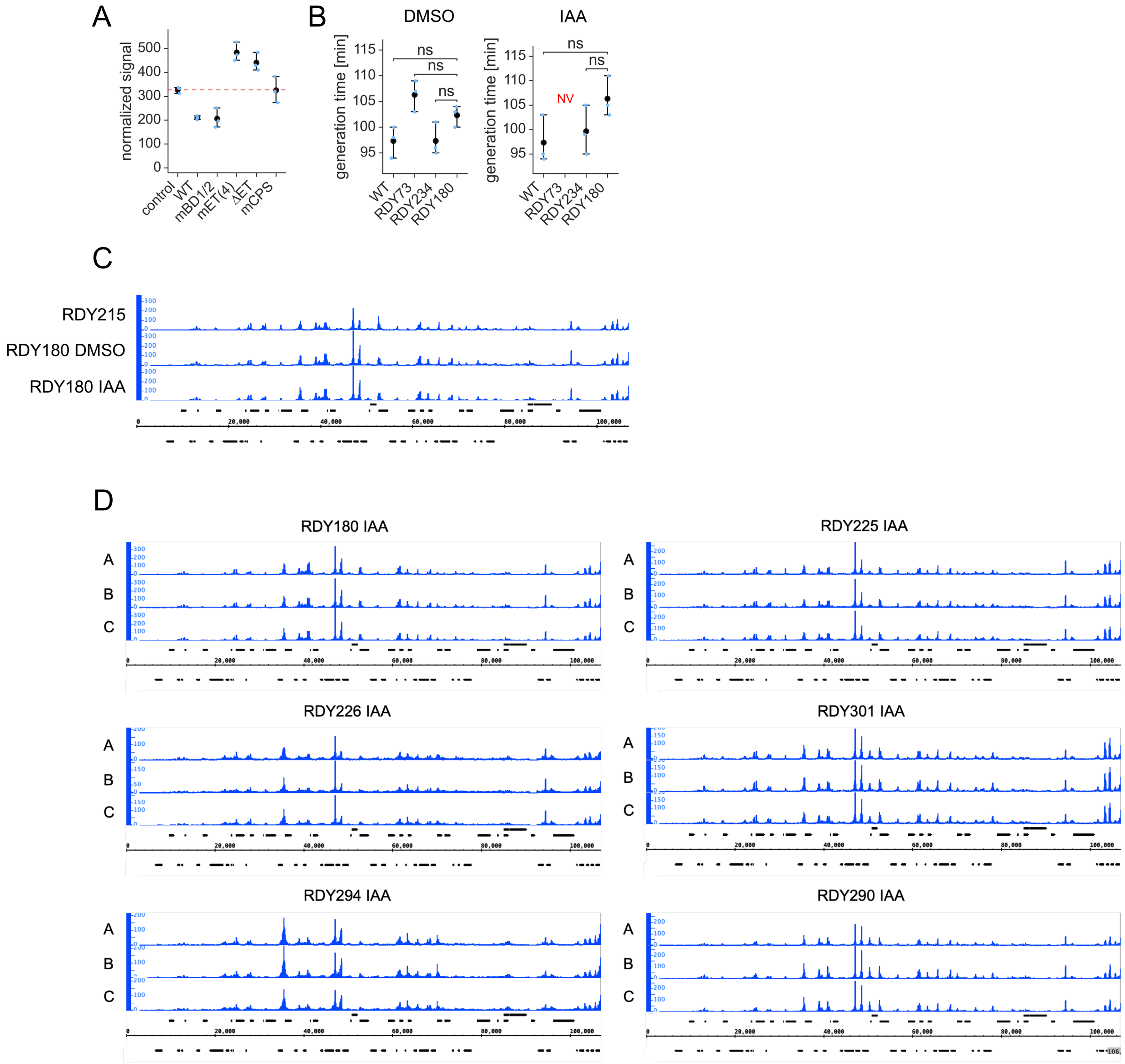

Fig. S5. ChEC-seq allows reproducible mapping of chromatin occupancy for ectopically expressed Bdf1 variants. (**A**) Western blot analysis of the expression of the indicated *BDF1-MNase* fusion variants. *BDF1-MNase* variants were expressed from the *TRP1* locus of strain RDY73 without depletion of endogenous Bdf1/2. The reference expression level (red dashed line) corresponds to the control strain expressing unmodified Bdf1 from the endogenous locus. An anti-Bdf1 antibody was used (a gift from Stephen Buratowski). Fluorescent signal for total protein content (LI-COR total protein stain workflow) was used to normalize target proteins. Black markers indicate mean normalized signal. Error bars represent the 95% confidence interval (n = 3). (**B**) Growth assay comparing the fitness of a laboratory wild-type strain (BY4705, WT), the RDY73 strain, the RDY234 strain (unmodified *BDF1* expressed from the *TRP1* locus) and a RDY73 derivative strain expressing a *BDF1-MNase* fusion protein from the *TRP1* locus (RDY180). The data were collected with (IAA) or without (DMSO) depletion of endogenous Bdf1/2, as indicated. Black markers indicate mean generation time. Error bars correspond to the 95% confidence interval (n = 3). Results of a Welch's t-test are shown (ns – not significant). NV indicates an inviable strain (RDY73 in IAA conditions). (**C**) Genome browser

snapshot comparing a binding pattern of ectopically expressed Bdf1 in strain RDY180 in the presence (DMSO) or absence (IAA) of endogenous Bdf1/2 to that of the reference strain RDY215. Data for the first 100,000 bases of chromosome III is shown**.** (**D**) Comparison of ChEC-seq data for individual replicate experiments for selected strains. Data for the first 100,000 bases of chromosome III is shown.

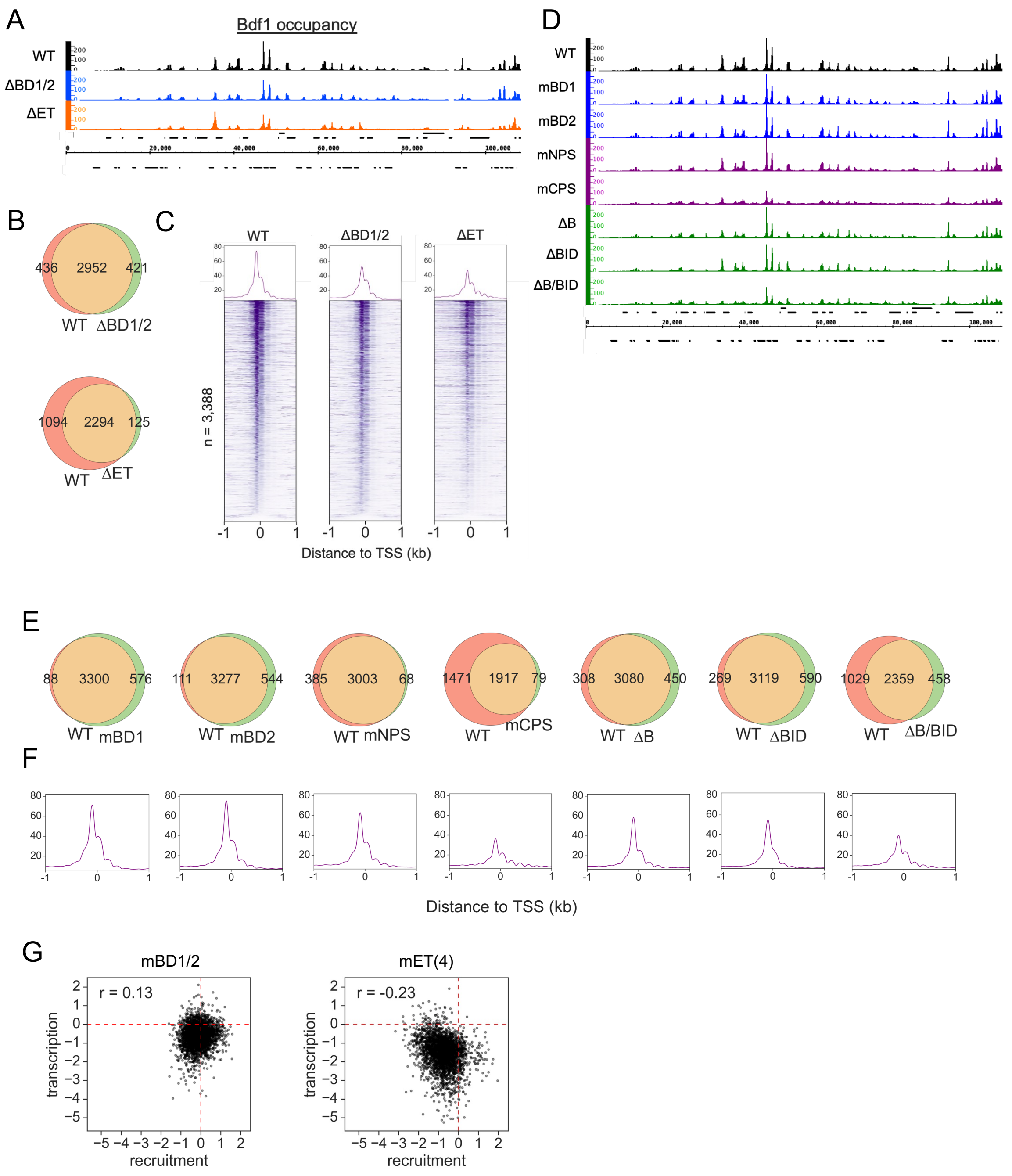

Fig. S6. CPS and B/BID regions are important for Bdf1 chromatin occupancy. (**A**) Genome browser snapshot showing the binding pattern of unmodified Bdf1 (WT) and Bdf1 variants with deletions of the BDs or the ET domain as determined by ChEC-seq. Data for the first 100,000 bases of chromosome III is shown. (**B**) Overlap of Bdf1-bound promoters for the indicated ChEC-seq experiments. (**C**) Occupancy of unmodified Bdf1 and Bdf1 variants with mutations in the BDs or the ET domain around the transcription start sites (TSS) of 3,388 genes whose promoters are bound by unmodified Bdf1. Genes are sorted by the TSS signal (+/- 200 bp) in the WT experiment. (**D**) Genome browser snapshot showing binding pattern of unmodified Bdf1 (WT) and the indicated Bdf1 variants as determined by ChEC-seq. Data for the first 100,000 bases of chromosome III is shown. (**E**) Overlap of Bdf1-bound promoters for the indicated

experiments. (**F**) Average occupancy of the indicated Bdf1 variants around the transcription start sites (TSS) of 3,388 genes whose promoters are bound by unmodified Bdf1**.** (**G**) Comparison of changes in Bdf1 promoter occupancy due to mutations in the BDs or the ET domain and corresponding changes in transcription. Pearson correlation coefficient (r) is shown.

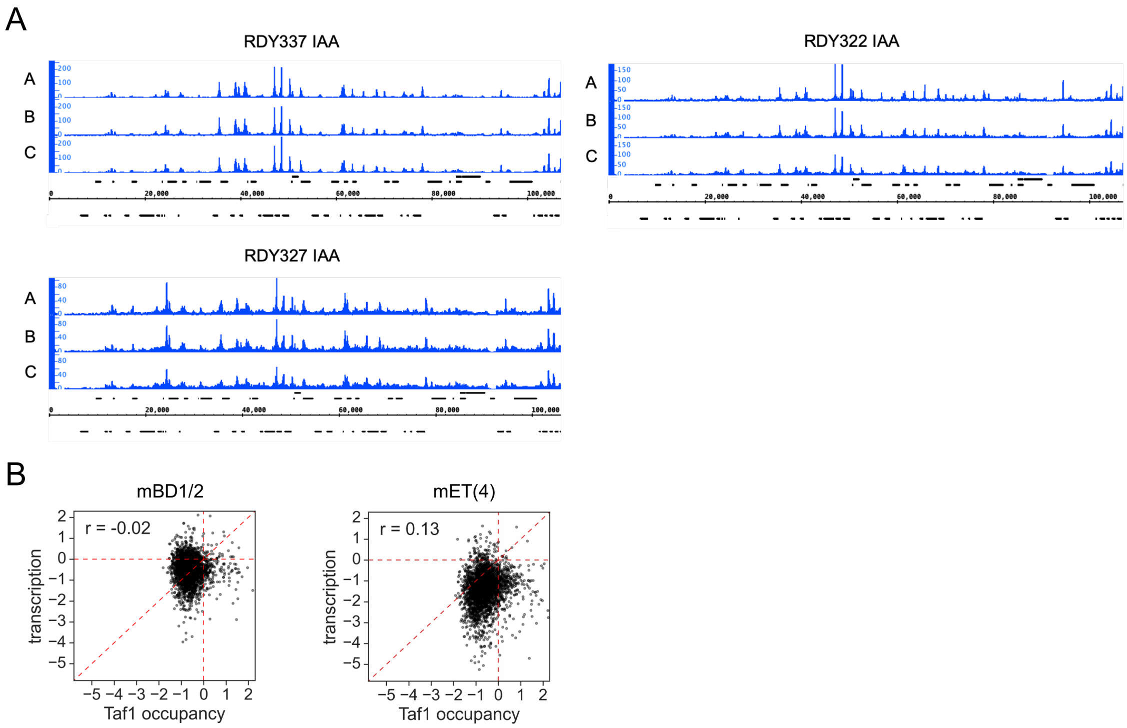

Fig. S7. The ET domain facilitates recruitment of TFIID to promoters. (**A**) Comparison of ChEC-seq data for individual replicate experiments. Data for the first 100,000 bases of chromosome III is shown. (**B**) Comparison of changes in Taf1 promoter occupancy due to mutations in the BDs or the ET domain and corresponding changes in transcription. Pearson correlation coefficient (r) is shown.

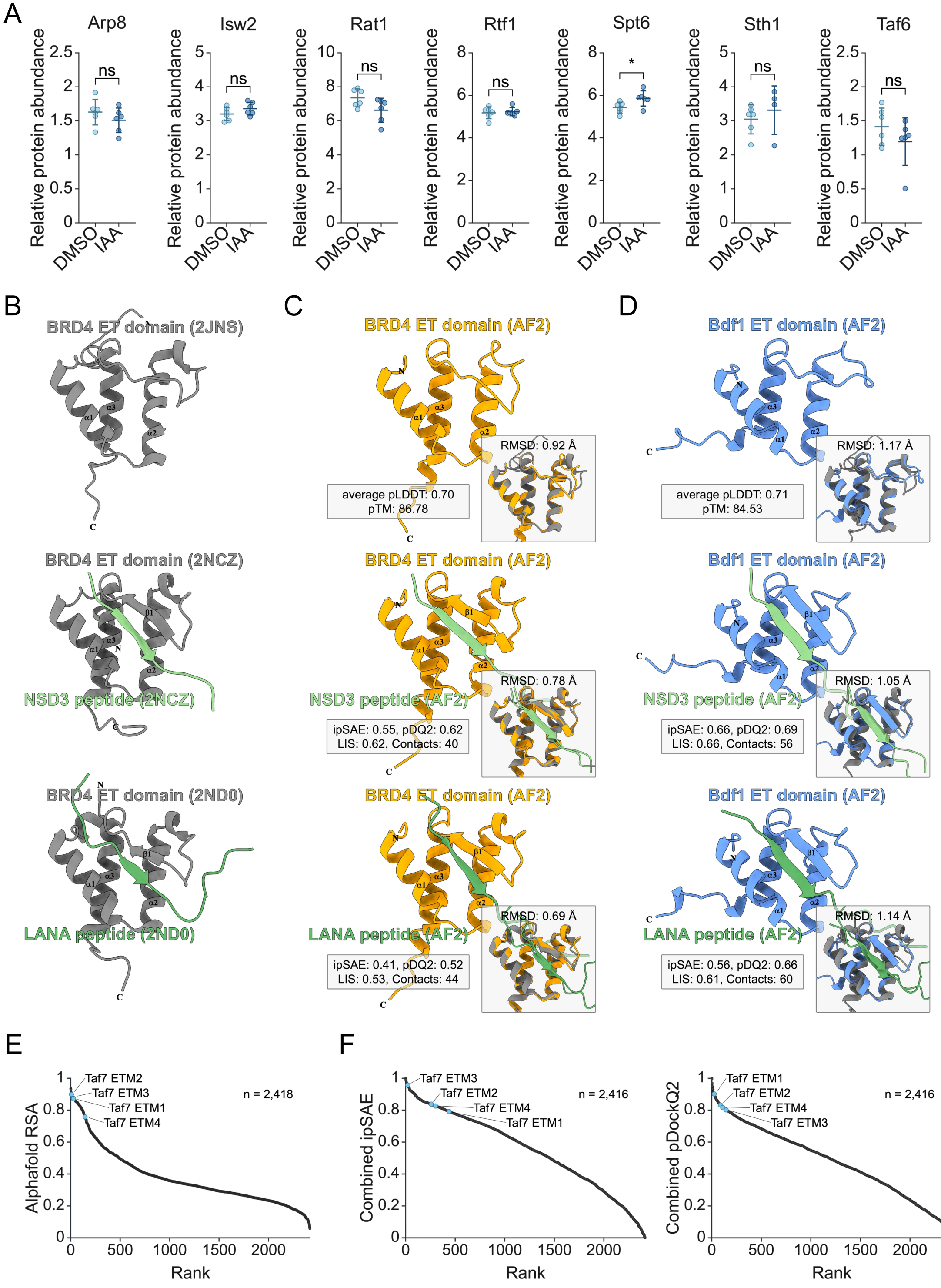

Fig. S8. Protein levels are largely stable in whole cell extract following acute depletion of Bdf1/2, and structural predictions for the BRD4 or Bdf1 ET domains in association with ETMs are highly similar to experimental structures. (**A**) Relative protein abundance in whole cell extract for selected proteins following Bdf1/2 depletion for 25 min as determined by chromatin mass spectrometry. Selected proteins are highlighted in **Fig 5A**. Taf2 and Rsc2 were

not detected in whole cell extracts. Central lines indicate mean relative protein abundance. Error bars correspond to standard deviation of the mean (n = 6). Results of a Welch's t-test are shown (* – p-value < 0.05, ns – not significant). (**B**) Previously determined experimental structures for the mouse BRD4 ET domain alone (PDB: 2JNS; (81)) (top panel) or the human BRD4 ET domain in association with characterized ETMs from NSD3 (PDB: 2NCZ (24)) (middle panel) or LANA (PDB: 2ND0 (24)) (bottom panel). (**C**) AlphaFold2 (AF2) predicted structures for the human BRD4 ET domain alone (top panel) or in association with ETMs from NSD3 (middle panel) or LANA (bottom panel). Confidence metrics (ipSAE, pDockQ2, and LIS) and the number of interfacial contacts supporting structural predictions are shown. Insets compare predicted and experimental structures with corresponding RMSD values. (**D**) AlphaFold2 (AF2) predicted structures for the Bdf1 ET domain alone (top panel) or in association with ETMs from NSD3 (middle panel) or LANA (bottom panel). Confidence metrics (ipSAE, pDockQ2, and LIS) and the number of interfacial contacts supporting the structural predictions are shown. Insets show comparisons between predicted and experimental structures and RMSD supporting the comparisons. (**E**) Scatter plot comparing Alphafold RSA scores and rank order for ETMs in nuclear proteins (n = 2,418). Four putative ETMs in the Bdf1-interaction region of Taf7 are shown in blue. (**F**) Scatter plots comparing Combined ipSAE scores (left panel) or Combined pDockQ2 scores (right panel) and rank order for structural predictions of the Bdf1 ET domain in association with ETMs in nuclear proteins (n = 2,416). Predicted structures involving four putative ETMs in the Bdf1-interaction region of Taf7 are shown in blue in both panels.

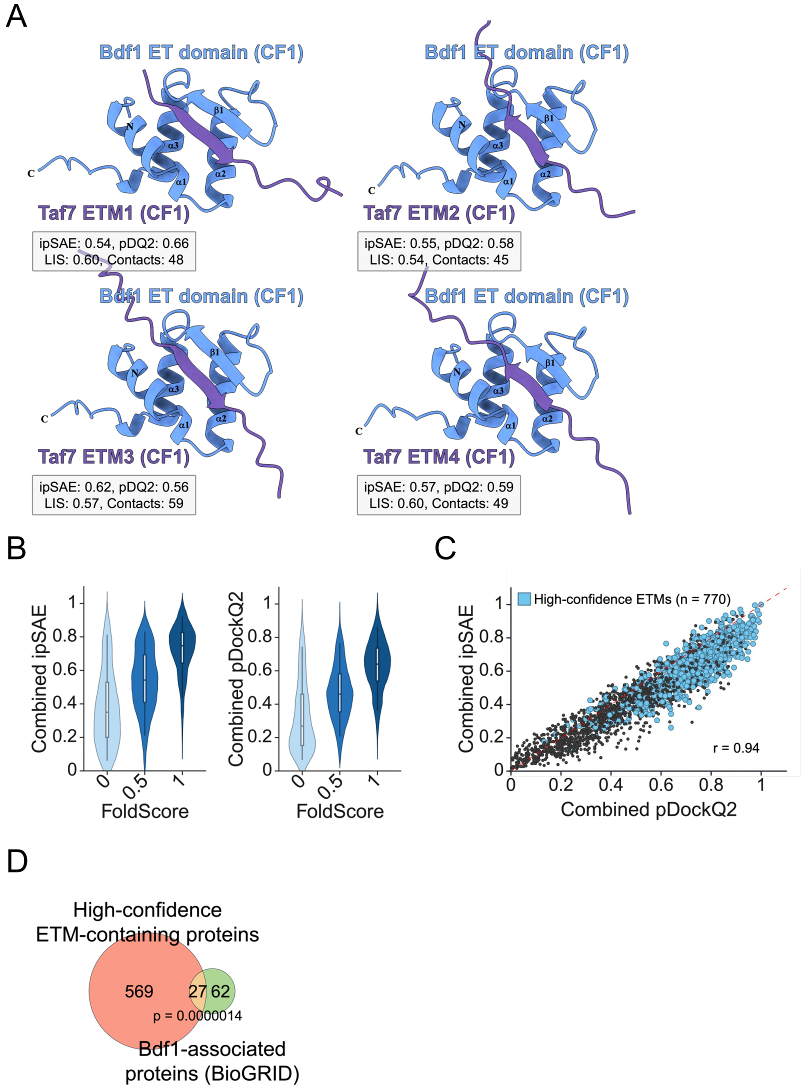

Fig. S9. High-confidence ETMs are correlated with conditional interfacial β-sheet establishment, and proteins carrying these ETMs are enriched for functions related to transcription regulation and reported association with Bdf1. (**A**) ColabFold (CF1) predicted structures for the Bdf1 ET domain in association with four putative ETMs in the Bdf1-interaction region of Taf7. Confidence metrics (ipSAE, pDockQ2, and LIS) and the number of interfacial contacts supporting structural predictions are shown. (**B**) Violin plots comparing Combined ipSAE scores (left panel) or Combined pDockQ2 scores (right panel) and FoldScore for structural predictions of the Bdf1 ET domain in association ETMs in nuclear proteins (*n* = 2,416). (**C**) Scatter plot comparing Combined ipSAE scores and Combined pDockQ2 scores for structural predictions of the Bdf1 ET domain in association with ETMs in nuclear proteins (*n* = 2,416). Structural predictions involving high-confidence ETMs (*n* = 770) are shown in blue. High-confidence ETMs have an AlphaFold RSA ≥ 0.24 and a FoldScore = 1. Pearson correlation coefficient (r) is shown. (**D**) Euler plot comparing high-confidence ETM-containing proteins (*n* = 770) and proteins associated with Bdf1. The set of Bdf1-associated proteins was obtained from BioGRID (https://www.thebiogrid.org). Proteins with evidence for physical association were selected. Proteins identified in affinity capture RNA experiments or in a high-

throughput Y2H study on coiled-coil domain interactions (82) were excluded. Results of a Fisher's exact test are shown.

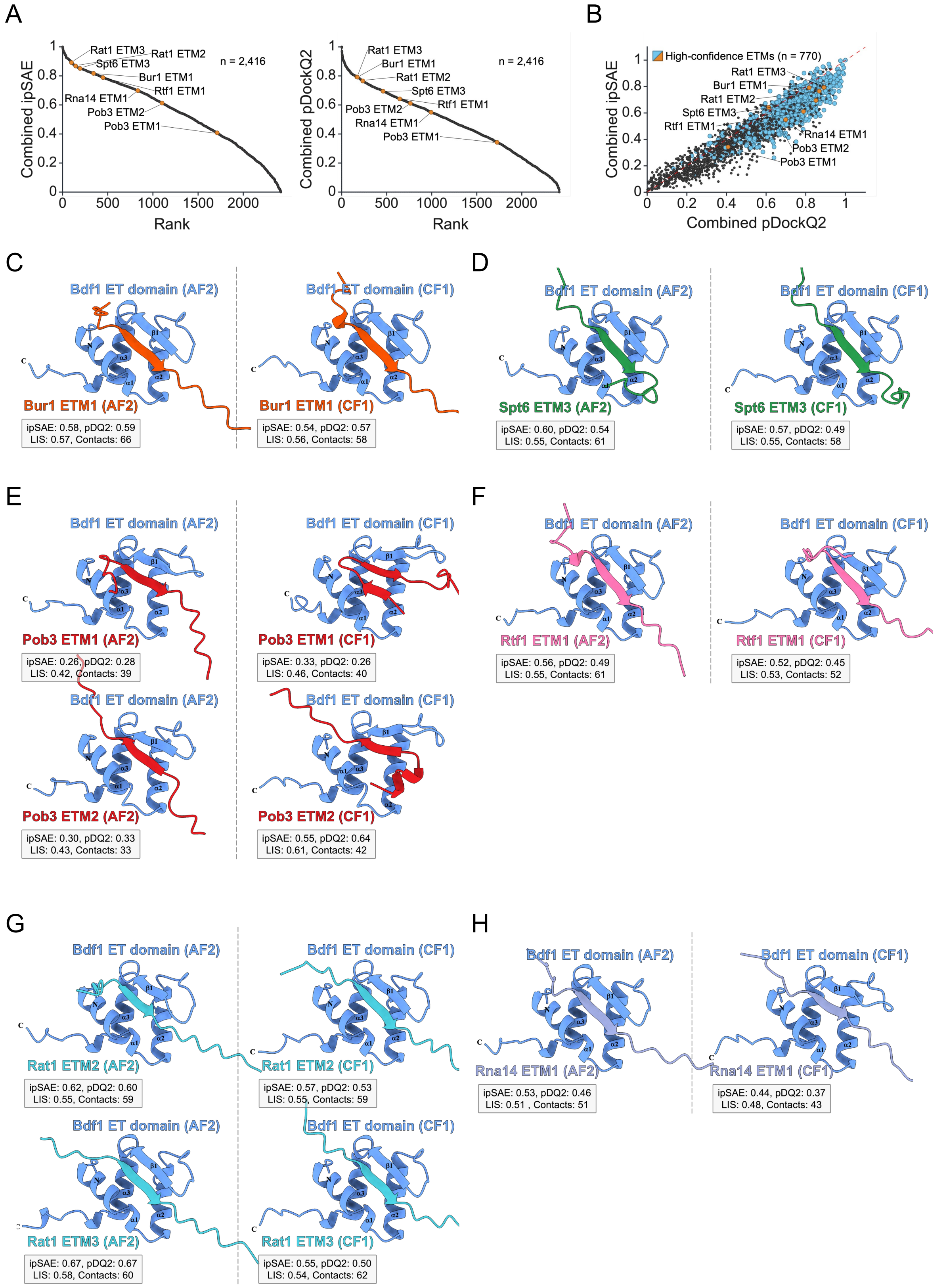

A
Combined ipSAE
Rank
n = 2,416
Rat1 ETM3
Rat1 ETM2
Spt6 ETM3
Bur1 ETM1
Rtf1 ETM1
Rna14 ETM1
Pob3 ETM2
Pob3 ETM1
Combined pDockQ2
B
High-confidence ETMs (n = 770)
C
Bdf1 ET domain (AF2)
Bdf1 ET domain (CF1)
Bur1 ETM1 (AF2)
Bur1 ETM1 (CF1)
ipSAE: 0.58, pDQ2: 0.59
LIS: 0.57, Contacts: 66
ipSAE: 0.54, pDQ2: 0.57
LIS: 0.56, Contacts: 58
D
Spt6 ETM3 (AF2)
Spt6 ETM3 (CF1)
ipSAE: 0.60, pDQ2: 0.54
LIS: 0.55, Contacts: 61
ipSAE: 0.57, pDQ2: 0.49
LIS: 0.55, Contacts: 58
E
Pob3 ETM1 (AF2)
Pob3 ETM1 (CF1)
ipSAE: 0.26, pDQ2: 0.28
LIS: 0.42, Contacts: 39
ipSAE: 0.33, pDQ2: 0.26
LIS: 0.46, Contacts: 40
Pob3 ETM2 (AF2)
Pob3 ETM2 (CF1)
ipSAE: 0.30, pDQ2: 0.33
LIS: 0.43, Contacts: 33
ipSAE: 0.55, pDQ2: 0.64
LIS: 0.61, Contacts: 42
F
Rtf1 ETM1 (AF2)
Rtf1 ETM1 (CF1)
ipSAE: 0.56, pDQ2: 0.49
LIS: 0.55, Contacts: 61
ipSAE: 0.52, pDQ2: 0.45
LIS: 0.53, Contacts: 52
G
Rat1 ETM2 (AF2)
Rat1 ETM2 (CF1)
ipSAE: 0.62, pDQ2: 0.60
LIS: 0.55, Contacts: 59
ipSAE: 0.57, pDQ2: 0.53
LIS: 0.55, Contacts: 59
Rat1 ETM3 (AF2)
Rat1 ETM3 (CF1)
ipSAE: 0.67, pDQ2: 0.67
LIS: 0.58, Contacts: 60
ipSAE: 0.55, pDQ2: 0.50
LIS: 0.54, Contacts: 62
H
Rna14 ETM1 (AF2)
Rna14 ETM1 (CF1)
ipSAE: 0.53, pDQ2: 0.46
LIS: 0.51 , Contacts: 51
ipSAE: 0.44, pDQ2: 0.37
LIS: 0.48, Contacts: 43

Fig. S10. Structural modeling supports associations between the Bdf1 ET domain and key factors in transcription regulation. (**A**) Scatter plots comparing Combined ipSAE scores (left panel) or Combined pDockQ2 scores (right panel) and rank order for predicted structures of the Bdf1 ET domain in association with ETMs in nuclear proteins (n = 2,416). Predicted structures involving high-confidence ETMs in Bur1, Spt6, Pob3, Rtf1, Rat1, or Rna14 are shown in orange in both panels (n = 8). (**B**) Scatter plot comparing Combined ipSAE scores and Combined pDockQ2 scores for structural predictions of the Bdf1 ET domain in association with ETMs in nuclear proteins (n = 2,416). Structural predictions involving high-confidence ETMs in Bur1, Spt6, Pob3, Rtf1, Rat1, or Rna14 are shown in orange (n = 8). Structural predictions involving the remaining high-confidence ETMs are shown in blue (n = 762). (**C-H**) Predicted structures for the Bdf1 ET domain in association with high-confidence ETMs in Bur1, Spt6, Pob3, Rtf1, Rat1, or Rna14. AlphaFold2 (AF2) and ColabFold (CF1) predicted structures are shown in right and left panels, respectively. Confidence metrics (ipSAE, pDockQ2, and LIS) and the number of interfacial contacts supporting structural predictions are shown.

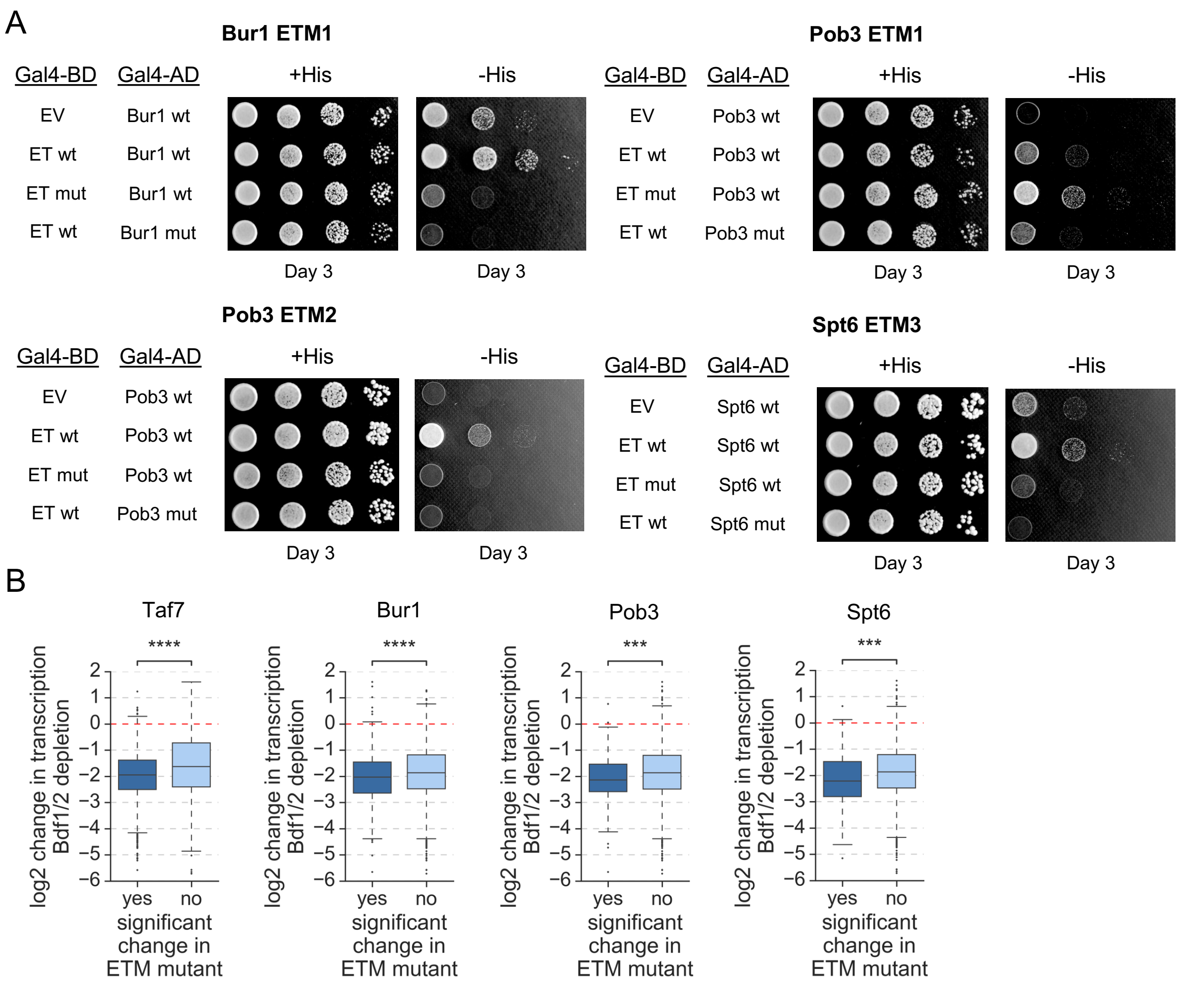

Fig. S11. The loss of interactions between the Bdf1 ET domain and ETMs in Bur1, Pob3, and Spt6 contributes to the overall defects observed with Bdf1/2 depletion. (**A**) Yeast two-hybrid analysis of interactions between the Bdf1 ET domain and high-confidence ETMs in Bur1, Pob3, and Spt6. Plasmids expressing the Bdf1 ET domain (WT or mut), fragments of Bur1, Pob3, or Spt6 that cover the indicated ETMs (WT or mut), or an empty vector (EV) were co-transformed into the host strain. Cells were serially diluted, spotted on the indicated media, and imaged after three days of growth (n = 3). ET mut – ET domain with four conserved acidic residues substituted with alanine. Basic residues in all ETM mutants are substituted with alanine. Amino acid coordinates of Bur1, Pob3, and Spt6 peptides used are as follows: Bur1 ETM1 – 2-62, Pob3 ETM1 – 239-383, Pob3 ETM2 – 381-552, Spt6 ETM3 – 1249-1451. (**B**) Log2 changes in transcription following depletion of Bdf1/2 for 25 min. Genes are categorized into two groups based on the statistical significance of the changes observed in relation to ETM mutations in the indicated factors (Taf7, Bur1, Pob3, or Spt6) shown in Fig. 6C. Results of a Welch's t-test are shown (*** – p-value < 0.001, **** – p-value < 0.0001).

Supplementary tables associated with this study:

Table S1. List of plasmids used in this study.

Table S2. List of *S. cerevisiae* strains used in this study.

Table S3. Results of SLAM-seq experiments (Figs. 2A-F, 6C, S2, S3, S4A-B, S4D-E and S11B).

Table S4. Log2 changes in Bdf1 promoter occupancy due to *BDF1* mutations (Figs. 3 and S6).

Table S5. Results of chromatin mass spectrometry experiments (Figs. 5A and S8A).

Table S6. Summary of ET-interacting motif (ETM) discovery (Fig. 5B). A list of 2418 ETMs identified in yeast nuclear proteome is shown in tab D.

Table S7. Summary of structural predictions and associated analysis (Figs. 5D, S8B-F, S9 and S10). Lists of high-confidence ETMs and associated proteins are shown in tabs E and F, respectively.
